# Commentary: Heads-up limit hold'em poker is solved

**DOI:** 10.3389/fpsyg.2018.00210

**Published:** 2018-02-21

**Authors:** Philip W. S. Newall

**Affiliations:** Technical University of Munich, Munich, Germany

**Keywords:** games, game theory, expertise, artificial intelligence, decision making

The game of poker, with its tactics of bluffing and deception, has frequently captured the imagination. In one example from popular culture, James Bond defeats a terrorist financier at the poker table in the film *Casino Royale*. Bond's poker skill reflects his abilities as a spy: Spotting lies and deception, and thinking one move ahead of his opponent. But like other domains of human skill, poker has been affected by the rise of the machines. In 2015, a supercomputer with 48 CPUs running for 68 days “solved” heads-up limit hold'em poker, the simplest poker game played for money in casinos and online (Bowling et al., 2015). This computer cannot be beaten, even in a human lifetime of play. This commentary analyzes the perfect strategy from Bowling et al.'s target article to ask: Does the computer's strategy in the game's key initial decision reflect poker expert wisdom, or does the computer play entirely differently?

Games are a common domain for testing the relative skills of experts and computers. In 1997 Garry Kasparov famously lost to Deep Blue in chess, and in 2016 Lee Sedol lost to AlphaGo. In 2008 a similar expert-computer match occurred for heads-up limit hold'em poker (CPRG, 2008). A team of seven professionals played “Polaris,” a computer designed by researchers from the University of Alberta (who later built the 2015 supercomputer). Polaris was the overall winner, although the professional Matt Hawrilenko, who was viewed by many as the most-skilled in this poker game (Brodie, 2008; Arnett, 2009; Nalbone, 2011), emerged a net winner.

Even a relatively simple card game involving two players and a 52 pack of cards can create significant complexity. More precisely, there are 3.16 × 10^17^ potential game states in this poker game (Bowling et al., 2015). Humans must initially simplify complex problems to learn and improve their performance (Dreyfus and Dreyfus, 1986). We use simple “heuristics” even for problems much simpler than this poker game (Gigerenzer et al., 1999; Hertwig et al., 2013). Poker theorists suggest two relevant simplifying principles: aggression and information hiding (Chen and Ankenman, 2006). It is generally better to be aggressive by *raising* the stakes, rather than equalling the stakes by *calling*. It is generally better to hide information by playing many hands the same way, rather than having a unique strategy for specific hands.

Here is a simple strategy as the first player on the first round reflecting these principles (this situation is both important and relatively simple to analyze). This player can play any individual hand by *folding* (putting no more money in, and immediately forfeiting the hand), *calling* (equalling the bet), or by *raising* (doubling the bet). The strategy involves finding a single threshold point: All hands weaker than this are *folded*, and those stronger are played by *raising* (*raise-or-fold*). *Calling*, as a potential strategy, is never considered (on the initial first round decision; calling might be done later on). Now, the first player's first round strategy must specify play in one additional scenario. If the second player *re-raises*, then the first player is revisited with the fold, call, raise trilemma. (If the second player folds the hand immediately ends; if the second player calls play moves onto the second round.) In this case folding is inadvisable from a risk-reward perspective (Sklansky, 1999). A similar argument means raising accomplishes little (because a skilled second player will never fold). Therefore, *always-calling* is the recommended simple strategy (information hiding trumps aggression in this instance where the principles conflict). So the first player should *raise-or-fold* (based on initial hand strength), and then *always-call*. The entire first round strategy boils down to a single hand: The worst hand worth raising. An effective strategy could not be simpler.

Matt Hawrilenko followed this strategy in the 2008 match (Newall, 2011). Over 1,000 hands, he raised 86.8%, otherwise folding. When facing a re-raise he called every time. Computers do not face the same computational constraints as humans. So it is perhaps not surprising that Polaris, the 2008 computer, used a similar yet more-complex strategy. Polaris raised 85.0% (1.8% lower than Hawrilenko), but called 2.4% (rather than never). When facing a re-raise, Polaris called 83.6% of the time, otherwise raising (compared to Hawrilenko calling 100%).

Polaris from 2008 is significantly weaker at poker and used fewer computational resources than “Cepheus,” the unbeatable 2015 agent (Bowling et al., 2015). So how does Cepheus compare? Surprisingly, the more complex computer agent actually uses a simpler strategy. Table 1 compares the three strategies' observable behavior (combining data from Newall, 2011; Bowling et al., 2015). Cepheus initially raises 82.54%, only calling a miniscule 0.06%. Cepheus's initial calling frequency is closer to Hawrilenko's than Polaris's. Cepheus calls 99.1% when facing a re-raise, again much closer to Hawrilenko than Polaris. But you would not be recommended to copy these rare plays. According to one of the study's co-authors, Cepheus's deviations from Hawrilenko's simpler strategy are, “most likely part of the noise that makes it ‘essentially’ solved and not just solved” (Burch, 2015).

**Table 1:** Action frequencies as the first player.

**Action**	**Matt Hawrilenko (2008)**	**Polaris (2008)**	**Cepheus (2015)**
**INITIAL DECISION**			
Fold	13.2%	12.6%	17.4%
Call	0%	2.4%	0.06%
Raise	86.8%	85.0%	82.54%
**WHEN FACING A RE-RAISE**			
Fold	0%	0%	0%
Call	100%	83.6%	99.9%
Raise	0%	16.4%	0.1%

In conclusion, the expert's strategy in 2008 in this key situation closely matched the unbeatable 2015 computer's strategic frequencies. This is another example of expert performance approaching perfect game theoretic strategy (Walker and Wooders, 2001; Chiappori et al., 2002; Palacios-Huerta, 2003). Although the 2015 computer is unbeatable, the expert's knowledge is more robust to other poker games (Lake et al., 2016). And the expert can adjust strategy to take greater advantage of opponents' mistakes. (Newall, 2013, explores less crucial situations in this poker game where computers play unlike most experts.) Simple heuristics have been recommended for changing environments (Bookstaber and Langsam, 1985), for when computational resources are limited (Simon, 1955), and when effort must be economized (Shah and Oppenheimer, 2008). Yet it appears that the supercomputer's optimization led it to a simple strategy like the expert's, even when none of these arguments applied (Parpart et al., 2017).

## Author contributions

The author confirms being the sole contributor of this work and approved it for publication.

### Conflict of interest statement

The author declares that the research was conducted in the absence of any commercial or financial relationships that could be construed as a potential conflict of interest.

## References

[B1] Arnett K. (2009). A Poker Life – Matt Hawrilenko. Available online at: http://www.cardplayer.com/poker-news/7826-a-poker-life-matt-hawrilenko

[B2] Bookstaber R. Langsam J. (1985). On the optimality of coarse behavior rules. J. Theor. Biol. 116, 161–193. 10.1016/S0022-5193(85)80262-9 4058019

[B3] Bowling M. Burch N. Johanson M. Tammelin O. (2015). Heads-up limit hold'em poker is solved. Science 347, 145–149. 10.1126/science.1259433 25574016

[B4] Brodie R. (2008). A Heads-Up for Human Poker Players. Available online at: http://www.liontales.com/2008/07/

[B5] Burch N. (2015). Re: Computers Conquer Texas Hold'em Poker for First Time. Available online at: http://forumserver.twoplustwo.com/showpost.php?p=45771353&postcount=31

[B6] Chen B. Ankenman J. (2006). The Mathematics of Poker. Pittsburgh, PA: ConJelCo LLC.

[B7] Chiappori P. Levitt S. Groseclose T. (2002). Testing mixed-strategy equilibria when players are heterogeneous: the case of penalty kicks in soccer. Am. Econ. Rev. 92, 1138–1151. 10.1257/00028280260344678

[B8] CPRG (2008). The Second Man-Machine Poker Competition. Available online at: http://webdocs.cs.ualberta.ca/~games/poker/man-machine/

[B9] Dreyfus H. L. Dreyfus S. E. (1986). Mind Over Machine. New York, NY: The Free Press.

[B10] Gigerenzer G. Todd P. M. The ABC Research Group (1999). Simple Heuristics That Make Us Smart. New York, NY: Oxford University Press.

[B11] Hertwig R. Hoffrage U. The ABC Research Group (2013). Simple Heuristics in a Social World. New York, NY: Oxford University Press.

[B12] Lake B. M. Ullman T. D. Tenenbaum J. B. Gershman S. J. (2016). Building machines that learn and think like people. arXiv:1604.00289. 27881212 10.1017/S0140525X16001837

[B13] Nalbone J. (2011). Princeton Grad Made Millions Playing Online Poker Until the Feds Upped the Ante. Available online at: http://www.nj.com/mercer/index.ssf/2011/06/princeton_grad_was_making_a_fi.html

[B14] Newall P. (2011). The Intelligent Poker Player. Las Vegas, NV: Two Plus Two Publishing.

[B15] Newall P. (2013). Further Limit Hold'em: Exploring the Model Poker Game. Las Vegas, NV: Two Plus Two Publishing.

[B16] Palacios-Huerta I. (2003). Professionals play minimax. Rev. Econ. Stud. 70, 395–415. 10.1111/1467-937X.00249

[B17] Parpart P. Jones M. Love B. C. (2017). Heuristics as Bayesian Inference under Extreme Priors. Available online at: https://psyarxiv.com/qkbt5 10.1016/j.cogpsych.2017.11.006 PMC5886040 29500961

[B18] Shah A. K. Oppenheimer D. M. (2008). Heuristics made easy: an effort-reduction framework. Psychol. Bull. 134, 207–222. 10.1037/0033-2909.134.2.207 18298269

[B19] Simon H. A. (1955). A behavioral model of rational choice. Q. J. Econ. 69, 99–118. 10.2307/1884852

[B20] Sklansky D. (1999). The Theory of Poker. Las Vegas, NV: Two Plus Two Publishing.

[B21] Walker M. Wooders J. (2001). Minimax play at wimbledon. Am. Econ. Rev. 91, 1521–1538. 10.1257/aer.91.5.1521

